# Keratins 6, 16, and 17 in Health and Disease: A Summary of Recent Findings

**DOI:** 10.3390/cimb46080508

**Published:** 2024-08-06

**Authors:** Daniil D. Romashin, Tatiana V. Tolstova, Alexandra M. Varshaver, Peter M. Kozhin, Alexander L. Rusanov, Natalia G. Luzgina

**Affiliations:** Institute of Biomedical Chemistry, Moscow 119121, Russia; dromashin@ibmc.msk.ru (D.D.R.); tolstova@ibmc.msk.ru (T.V.T.); ng-luzgina@mail.ru (N.G.L.)

**Keywords:** keratins, keratin 17, keratin 16, keratin 6, psoriasis, cancer, skin, wound healing

## Abstract

Keratins 6, 16, and 17 occupy unique positions within the keratin family. These proteins are not commonly found in the healthy, intact epidermis, but their expression increases in response to damage, inflammation, and hereditary skin conditions, as well as cancerous cell transformations and tumor growth. As a result, there is an active investigation into the potential use of these proteins as biomarkers for different pathologies. Recent studies have revealed the role of these keratins in regulating keratinocyte migration, proliferation, and growth, and more recently, their nuclear functions, including their role in maintaining nuclear structure and responding to DNA damage, have also been identified. This review aims to summarize the latest research on keratins 6, 16, and 17, their regulation in the epidermis, and their potential use as biomarkers in various skin conditions.

## 1. Introduction

Keratins are a superfamily of intermediate filamentous proteins that play important roles in the organization of the cytoskeleton. The cytoskeleton of mammalian epithelial cells is formed from pairs of type I and type II keratins. Type I keratins usually have a lower mass (40–56.5 kDa) and a more acidic total charge (pI 4.5–6.0), while type II keratins have a larger mass (50–70 kDa) and a basic neutral charge (pI 6.5–8.5) [1]. The heterodimers of keratin types I and II self-assemble into antiparallel, staggered tetramers, forming intermediate filaments through longitudinal and lateral interactions [2]. These keratin intermediate filaments (KIFs) have a diameter of approximately 10 nanometers and form a network within the cytoskeleton of cells, providing structural support and mechanical resilience.

The patterns of keratin expression in different tissues are not universal and mostly characterize specific types of epithelia. For example, keratins 8 and 18 are expressed in the simple epithelium, along with additional keratins 7, 19, 20, and 23. In contrast, stratified epithelium is characterized by keratins K4/K13 in mucous membranes and keratins K3/K12 in the cornea (reviewed by Kalabusheva et al. [3]). In the epidermis, actively proliferating basal keratinocytes express high levels of K5/K14 and low levels of K15. During epidermal differentiation, the proliferation of keratinocytes decreases, and keratins 5 and 14 are replaced by keratins 1 and 10 as the cells migrate to higher layers of the epidermis [4,5,6].

Moreover, the expression of certain keratins in the epidermis is highly restricted to specific anatomical sites. Keratin K6 (*KRT6*) and its polymerization partners K16 and K17 (*KRT16* and *KRT17*, respectively) are only constitutively expressed in the palmoplantar epidermis as well as in skin appendages, including the nail plate, hair follicles, and sebaceous and sweat glands [2,7,8]. Keratins K6/16/17 are not detected in intact normal interfollicular epidermis; however, their expression significantly increases in wounds and is maintained at a high level throughout all stages of regeneration until the complete restoration of epithelial barrier function [2,9,10]. At the same time, the expression of K1/K10 rapidly decreases in keratinocytes at the wound’s edge, and K6/K16 induction and dimerization occur, accompanied by an increase in the level of cytoplasmic keratin K17 [10]. K6, K16, and K17 are known as damage-associated keratins, and they are often referred to as “stress keratins” because they play a central role in the process of damage regeneration.

High expression of these keratins has been detected in hyperproliferating keratinocytes in psoriasis and other inflammatory skin conditions [2,11,12]. In addition, in clinical settings, keratin K17 is well known as an oncogenic protein, the high expression of which is often linked to a poor prognosis. Additionally, an increasing number of recent studies have focused on the roles of keratins K16 and K6 in relation to carcinogenesis and tumor progression [13,14,15].

The study of molecular physiological processes regulated by keratin 6/16/17 has revealed many functions that are atypical for these proteins. These include the regulation of cell migration, growth, proliferation, apoptosis (programmed cell death), nuclear morphology, and responses to DNA damage. This review aimed to summarize the latest research data on the biological functions of these proteins, their encoded genes, and their roles under normal and abnormal conditions. Their potential as clinical markers and targets for therapy are also discussed.

## 2. Structure and Localization of K6/K16/K17

**Figure 1:**
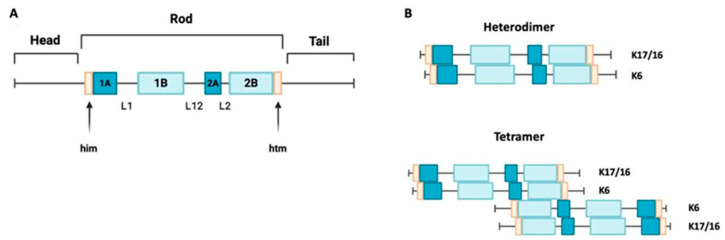
Keratin 17 structure and assembly. (**A**) A schematic structure of keratin 17 showing non-helical domains at the N-terminus and C-terminus ends, with a central helical rod domain. K17’s structure consists of four alpha-helical domains (1A, 1B, 2A, and 2B), separated by three non-helical linkers (L1, L12, and L2). K17 also has two motifs, the helix initiation (him) and termination (hit) motifs, located at the ends of domains 1A and 2B, respectively. (**B**) A depiction of the alignment of the acidic type I keratins K17 and K16 along with the basic keratin K6, arranged in parallel to create a heterodimer. These heterodimers align in an antiparallel and staggered fashion to form a tetramer.

Keratin 17 is a type I keratin with a molecular weight of 48 kDa. It has a triplet structure consisting of 432 amino acids. Its structure (Figure 1A) includes a nonhelical head region (1–83), an alpha-helical rod region (84–392), and a nonhelical tail region (393–432) [16,17]. Within the core domain, there are segments containing heptadic repeats (coils 1A, 1B, and 2), which are interrupted by linker sequences at two conserved sites. Keratin 16 is also a type I keratin with a molecular weight of 50,924.66 Da. It is structurally similar to keratin 17, but it is slightly larger, consisting of 473 amino acids [1]. A mini family of three genes (*KRT6A*, *KRT6B*, and *KRT6C*) encodes homologous intermediate filaments of type II. The protein encoded by the *KRT6A* gene is dominant in this family. All genes in this mini family encode proteins with 564 amino acids, but the calculated molecular weights of these proteins vary slightly [9].

Keratins 16 and 17 can form dimers with keratin 6 (Figure 1B), so the expression of these intermediate filaments is often colocalized [18]. To investigate this further, data from the Genotype-Tissue Expression (GTEX) database were analyzed in a recent study. The goal was to reconstruct the tissue-specific expression patterns of keratins in the human body. This study confirmed the co-expression of several pairs of keratin genes, including *KRT6* and *KRT16*, as well as *KRT6* and *KRT17* [19].

These data are in line with the fact that keratins K6, K16 and K17 are normally co-expressed in the palmar and plantar epidermis, as well as in epithelial appendages such as hair follicles. They are also found in epidermal lesions and in various pathological skin conditions [2,20,21]. In hair follicles, K6/K16/K17 are localized in the cells of the outer root sheath and in the cells of the companion layer and medulla [3,22,23,24].

In addition to the skin and its derivatives, K17 is expressed in normal basal cells of the respiratory epithelium, urothelial cells, squamous epithelial cells of the mucosal membrane, and basal cells of the oral cavity epithelium [10,25,26,27,28]. Keratin K16 has been detected at the protein level in various epithelial tissues and in nonkeratinizing stratified squamous epithelia [25,29,30]. K6 and K16 are also expressed in the duct cells of the mammary glands and sweat glands [31]. It has been reported that keratin 6 is expressed in the multilayered epithelium of the mucous membranes of the oral cavity, esophagus, and glandular epithelium [3,23,25]. However, given its colocalization with keratins K16 and K17, it is reasonable to assume that K6 is expressed in all types of epithelial cells expressing K16 and K17.

## 3. K6, K16, and K17 Are Essential for Regeneration

Proliferation is the final stage of skin wound healing, during which tissue integrity is restored due to cell division. At the same time, keratinocytes, which are the cells that make up the outermost layer of the skin, fill the damaged area by migrating and proliferating and participate in restoring the epidermal barrier through epidermal differentiation. During the healing process, keratinocytes near the wound temporarily suspend epidermal differentiation and undergo changes in protein translation, cell size, shape, and contact with other cells and the extracellular matrix, preparing for active migration and proliferation. Immediately after injury, keratinocytes at the edge of the wound significantly reduce the expression of keratin K1/K10 and induce the expression of keratins K6, K16, and K17 [2,32,33,34]. The high expression of keratins K6, K16, and K17 provides keratinocytes with the mechanical resilience required for migration and structural support [35]. However, the role of these keratins in regeneration extends beyond their structural function. The modulation of cell migration and adhesion to substrates is critical for successful regeneration and is an essential physiological function of keratins K6, K16, and K17. To close the defect in the epidermis, keratinocytes at the wound edge must first weaken their adhesion to each other and the basement membrane. This process is facilitated by alterations in the composition of the keratin cytoskeleton and desmosomes, which are considered the main factors determining adhesive strength. Wound healing is accompanied by a temporary reduction in desmosome adhesion, while keratin expression increases. Keratins 5/14 have been shown to contribute to the stabilization of desmosomes, while keratins K6/K17, on the contrary, lead to the destruction of desmosomes through the induction of protein kinase C and subsequent destabilization of epithelial layers [36]. Keratin 6 is able to interact directly with myosin II, which promotes cell adhesion and provides additional resistance to mechanical stress [37,38]. In addition, keratin 6 is expressed in cells at the edge of a wound, where it plays a role in regulating the collective migration of keratinocytes by inhibiting Src [39]. It also regulates cell–matrix interactions and intercellular adhesion [37]. Notably, keratinocytes isolated from *KRT6A*-/*KRT6B*-mice exhibit increased migration [38]. It has been shown in HaCaT keratinocytes that overexpression of keratin 16 leads to a decrease in migration activity. This decrease can be partially reversed by co-transfecting K6A [40]. In contrast, keratin 17 enhances the migratory activity of keratinocytes, as shown by data obtained from HaCaT cells in a scratch wound assay. The knockdown of *KRT17* results in a decrease in the rate of diabetic wound healing in mouse skin [33]. Keratin 17 promotes damage repair by inducing cell proliferation through STAT3 [17] and modulates lipid metabolism in keratinocytes to restore epidermal barrier functions [41]. Generally, keratins K6/16 provide optimal intercellular adhesion and collective migration for damage repair, while keratin 17 induces cell migration and proliferation.

## 4. K6, K16, and K17 Are Involved in the Pathogenesis of Various Skin Diseases

### 4.1. Psoriasis

The involvement of K17 in the development of psoriasis was first identified in 1990 [42]. In 1995, Leigh and his colleagues discovered that K17 can be found in the upper layers of psoriatic skin both in vivo and in vitro [11]. This discovery formed the basis for further research in this area. Since then, the understanding of K17 as a marker for psoriasis has increased, and studies have shown that its level correlates with the severity of the condition [43]. The role of keratin 17 in the pathogenesis of this disease is primarily realized by inducing keratinocyte proliferation through the STAT3 and 14-3-3/mTOR pathways and modulating the immune response [44,45]. Keratin 17 is an autoantigen that stimulates the production of proinflammatory cytokines by T cells [44]. These cytokines, in turn, induce the expression of stress keratins. Interestingly, the a/a sequence (amino acids 102–116 and 188–196) of keratin 17 closely resembles that of the streptococcal M6 protein, a superantigen in psoriasis [44,46]. Keratins 6 and 16 are highly expressed in psoriatic plaques and are characteristic of psoriatic keratinocytes [47]. However, their role in the pathogenesis of psoriasis has been less well studied than that of keratin 17. Keratin 16 has also been shown to contribute to the hyperproliferation of keratinocytes in psoriasis, and silencing keratin 16 leads to a reduction in VEGF secretion by psoriatic keratinocytes [12]. Keratin 16 is also involved in regulating inflammation by modulating damage-associated molecular patterns (DAMPs) and cytokines through the mitogen-activated protein kinase (MAPK) and/or epidermal growth factor receptor (EGFR) pathways [48].

### 4.2. Atopic Dermatitis

Atopic dermatitis (AD), also known as eczema, is a common chronic inflammatory autoimmune skin disorder. Increased expression of *KRT6A* has been observed in patients with this condition. In keratinocytes from patients with AD, *KRT6A* appears to inhibit inflammation-induced apoptosis and suppress IL-17A expression, making the K6A/IL-17 pathway a potential target for treatment [49]. In addition, a recent study revealed a correlation between variations in the *KRT6A* gene (single nucleotide polymorphisms) and disease severity [50]. Although there is less evidence to support the role of keratin 16 in AD, recent research has shown that increased expression of *KRT16* may be a marker for hyperproliferative keratinocytes in AD, and *KRT16*’s expression is significantly reduced in patients treated with dupilumab for AD [51,52]. Although it is not yet clear whether keratin 17 plays a role in the development of AD, this condition is characterized by an increase in the expression of several cytokines that trigger the production of keratin 17 [53].

### 4.3. Pachyonychia Congenita

Pachyonychia congenita (PC) is a rare genetic disorder that is inherited in an autosomal dominant pattern. It is characterized by keratoderma in the palms and soles of the feet, nail dystrophy, cyst formation, hyperkeratosis of hair follicles, and leukokeratosis of mucous membranes. Hyperhidrosis is also a common symptom of this condition. Mutations in genes such as *KRT6A*, *KRT16*, and *KRT17* have been found in patients with PC [54,55,56]. In particular, the Asn92Asp mutation in the *KRT17* gene and the Leu130Pro mutation in *KRT16* (both of which are located in the helix initiation motif) have been linked to this condition. Additionally, mutations in *KRT6A* and *KRT6B* have been identified in some patients with PC [57,58]. Mutations of p.Tyr465His in exon 7 of *KRT6A* and p.Glu413Gln in exon 6 of *KRT16* have also been linked to PC [59].

To date, progress in PC treatment is still unsatisfactory, and many therapies are at different stages of clinical development. Naturally, keratin 6/16/17 have been identified as potential therapeutic targets for PC. Sirolimus (rapamycin), an mTOR inhibitor, has been shown to reduce the expression of keratin 6A in human keratinocytes and reduce the severity of PC symptoms, although it has some side effects [60,61]. Other approaches include the use of statins for Stat1 repression [62], botulinum toxin injections [63], and siRNA therapy [64,65]. These methods involve complex and painful medical procedures that are not perfect. Therefore, the development of new therapies for treating PC or reducing its symptoms is urgently needed. Since PC serves as a prototype for siRNA-based therapies, genome editing techniques such as CRISPR-based therapeutics could potentially be considered for future research.

### 4.4. Other Skin Pathologies

In addition to psoriasis and PC, keratin 17 is also involved in the development of other skin conditions, such as lichen planus (LP). The pathogenesis of these conditions shares some similarities, including increased expression of keratin 17. In lichen planus, this expression is triggered by the induction of interferon gamma (IFN-γ) [66]. Similarly, *KRT6A*, *KRT6B*, and *KRT6C* are increased in patients with LP [67]. Elevated levels of *KRT17* have been noted in patients with prurigo nodularis (PN), a chronic inflammatory dermatosis characterized by the presence of multiple hyperkeratotic papules [68]. In addition, in PN, there is increased expression of K6 in all layers of the skin with lesions, particularly in the spinous layer. Conversely, K16 has been detected only in the basal and lower suprabasal layers [69]. In another study, using single-cell RNA profiling, increased expression of the *KRT6A*, *KRT6B*, *KRT6C*, and *KRT16* genes significantly characterized PN keratinocytes [70]. Inflammatory skin diseases are likely to lead to the production of these keratins, as the expression of K6/K16/K17 is associated with skin damage and strongly upregulated by proinflammatory cytokines.

## 5. Cancer

For many types of cancer, keratin 17 can be used as a diagnostic, prognostic, and predictive marker. Its increased expression often correlates with tumor aggressiveness and has a negative prognosis [71,72,73,74,75]. According to the analysis of data from various types of cancer, based on The Gene Expression Omnibus (GEO), The Cancer Genome Atlas (TCGA), and The Human Protein Atlas (HPA), as well as TIMER2 and other sources, a high level of keratin 17 was noted in most malignant neoplasms compared to normal tissues [76,77]. In addition, high expression of keratin 17 has been correlated with tumor size, depth of invasion, and metastasis in gastric cancer [78]. It has also been found to determine the low level of response to therapy in patients with squamous cell carcinoma of the head and neck (HNSCC), especially in those treated with immune checkpoint blockades [79]. Keratin 17 has been reported to be a negative prognostic biomarker for several types of cancer, including squamous cell carcinoma of the lungs (LSCC) and pulmonary adenocarcinoma (LUAD) [80]. It has also been linked to pancreatic ductal adenocarcinoma (PDAC) [81] and colorectal cancer (CRC) [82]. Keratin 17 has been shown to induce and promote the growth of oral squamous cell carcinoma (OSCC) [83]. Although the expression of keratin 17 is generally associated with an unfavorable prognosis for cancer patients, this trend does not always hold true. For example, in invasive breast carcinoma (BRCA) and chromophobic kidney cancer (KICH), the levels of keratin 17 are lower than those in normal tissue. Additionally, there are no significant differences in the levels of keratin 17 between tumors and normal tissues in urothelial bladder carcinoma (BLCA), papillary carcinoma of the kidney (KIRP), or pancreatic adenocarcinoma (PAAD) [16].

The involvement of K6 and K16 in carcinogenesis and tumor progression has been studied to a lesser extent. However, the number of studies characterizing these oncoproteins has increased in recent years. Keratin 16, in particular, has been identified as a marker for circulating tumor cells. Its high expression in breast cancer samples has been correlated with an intermediate mesenchymal phenotype [14]. Huang and colleagues demonstrated that high levels of keratin 16 are associated with a poorer prognosis, pathological differentiation, and lymph node involvement in patients with oral squamous cell carcinoma (OSCC) [13]. Increased expression of keratin 6A and the involvement of keratin 16 in the development of this type of tumor have also been reported in LUAD [84]. For keratin 6, a correlation with squamous cell differentiation in As-transformed UROtsa cells has been shown [85]. In addition, increased expression of *KRT6A/B/C* has also been observed in primary melanoma cells, and high levels of these keratins have been associated with a poor prognosis for patients with skin cutaneous melanoma (SKCM) [20]. High levels of keratin 6A are associated with a poorer prognosis and increased invasiveness in colorectal cancer (CRC) [86]. Data on the diagnostic significance of K6, K16, and K17 proteins are summarized in Table 1.

## 6. The Role of K6, K16, and K17 in Carcinogenesis

Keratin 17 plays a role in the development of cancer through several key processes: (I) promoting cell growth and proliferation; (II) supporting cell survival; and (III) modulating the immune system’s response. Keratin 17 has been shown to be involved in regulating the proliferation, migration, and invasion of tumor cells. For example, it has been found to be important in the development of gastric cancer [78] and osteosarcoma [107], among other types of cancer. Thus, keratin 17 promotes cell proliferation and migration by activating Akt/mTOR [83,108], and its inhibition can lead to a reduction in the activity of ERK1/2, Bad, p38 MAPK, and SAPK/JNK [12]. Keratin 17 also contributes to the uncontrolled progression of the cell cycle by promoting the degradation of p27^KIP1^; therefore, K17 may be considered a marker for uncontrolled proliferation [109]. The discovery of the role of K17 in regulating the DDR response and cell survival suggests that one possible mechanism of carcinogenesis is the maintenance of cell viability with DNA damage. This, in turn, could contribute to the accumulation of mutations and genomic instability [110]. Suppression of keratin 17 expression can lead to the induction of tumor cell apoptosis through changes in the levels of Bcl-2 family proteins and enhanced regulation of caspase-3 cleavage. This contributes to stopping the cell cycle at the G1/S phase by reducing the expression of cyclin E1 and cyclin D [78]. Another important aspect of tumor progression is the role of keratin 17 in regulating glycolysis through its co-expression with HIF1α. When HIF1α is translocated to the nucleus, it binds to the promoters of specific target genes, such as GLUT1 [107]. In addition, keratin 17 covalently binds to α-enolase (ENO1), which supports its phosphorylation by K17-Ser44. This phosphorylation enhances the nuclear translocation of keratin 17 and the transcriptional activation of LDHA, leading to increased glycolysis and cell proliferation [8].

In addition, K17 plays a role in the process of tumor cells evading immune surveillance. CD8+ T cells have been shown to play a role in the recognition and induction of apoptosis in tumor cells, while K17 can block the infiltration of these cells into tumor tissues in some cancers. This has been demonstrated, in particular, in pancreatic cancer and proliferative processes associated with the papilloma virus [111]. At the same time, KRT17 knockdown slows down HNSCC growth and increases the infiltration of CD8+ T cells [103]. In addition, K17 suppresses macrophage-mediated CXCL9/CXCL10 chemokine signaling [112,113]. K17 also plays an oncogenic role by inducing the expression of IFN-γ, tumor necrosis factor alpha (TNF-α), and interleukin 10 (IL-10). These cytokines lead to the activation of different types of macrophages [114,115].

An increasing number of studies have investigated the functions of keratins 6 and 16 in cancer development and progression. One study revealed that silencing *KRT6A* led to a reduction in the expression of TIM-2 and MMP-9, two proteins involved in tumor invasion and metastasis in nasopharyngeal carcinoma [116]. A recent study revealed that KRT6A contributes to the progression of bladder cancer by enhancing cell viability, proliferation, and adhesion while also inhibiting cancer cell apoptosis [93]. In addition, activation of Notch1 in kidney cancer cells may lead to increased expression of *KRT6* [117]. *KRT6A* overexpression in LUAD promotes tumor cell proliferation, epithelial-to-mesenchymal transition (EMT), and metastasis [15]. Keratin 16 has also been reported to promote tumor progression and metastasis by inducing epithelial-to-mesenchymal transition (EMT), as has been demonstrated in LUAD and MCF-7 cells [14,84]. In addition, keratin 16 inhibits the degradation of vimentin, which can also promote metastasis [118]. A recent study showed that iRhom2 acts as a positive regulator of keratin 16. GOF mutations in iRhom2, which are found in tumors with esophageal cancer (TOC), support tumor hyperproliferation by modulating the expression of K16 [97].

## 7. Cell Functions of K6, K16, and K17

The most studied function of keratin 17 is its positive regulation of cell growth and proliferation. KRT17-deficient keratinocytes are smaller and exhibit reduced translation and suppressed expression of the mTOR/AKT signaling pathway, which is essential for regulating cell growth and synthesis [119]. In addition, keratin 17 promotes the phosphorylation of STAT3 and its nuclear transport, leading to the induction of cyclin D1 and the hyperproliferation of keratinocytes [17]. Phosphorylated keratin 17 regulates cell growth by binding to the adapter protein 14-3-3σ. In contrast, hypophosphorylation of keratin 17 leads to the sequestration of 14-3-3σ in the nucleus, inhibiting protein synthesis and cell growth [119]. Recent studies have revealed the functions of keratin 17 in the nuclear pool. This protein regulates the morphology of the nucleus, the structure of chromatin, RNA processing, and gene expression. It is also involved in regulating the DNA damage response by interacting with various proteins that are involved in DDRs, including Aire [120], hnRNPK [114], H2AX [121], DNA-PKcs [121], 53BP1 [121], and HMGN2 [110]. In addition, recent research has revealed that keratin 17 is essential for the overall regulation of gene expression. However, most of the mechanisms involved are still unknown [122].

The functions of keratin 16 have not been studied in detail, but it is known to play a role in regulating inflammation and innate immunity after damage to the skin barrier. Keratin 16 may influence the MAPK and EGFR signaling pathways, thereby regulating the overall levels of DAMPs [48]. K16 is also thought to be involved in protecting against oxidative stress through its interaction with the Nrf2 pathway. A recent finding was that keratin 16 helps maintain the normal redox balance in cells by activating Nrf2 and stimulating glutathione synthesis [123]. In addition, keratin 16 and its partner, keratin 6, are involved in regulating mitochondrial morphology, which could explain the signs of oxidative stress observed in PC patients [123].

**Figure 2:**
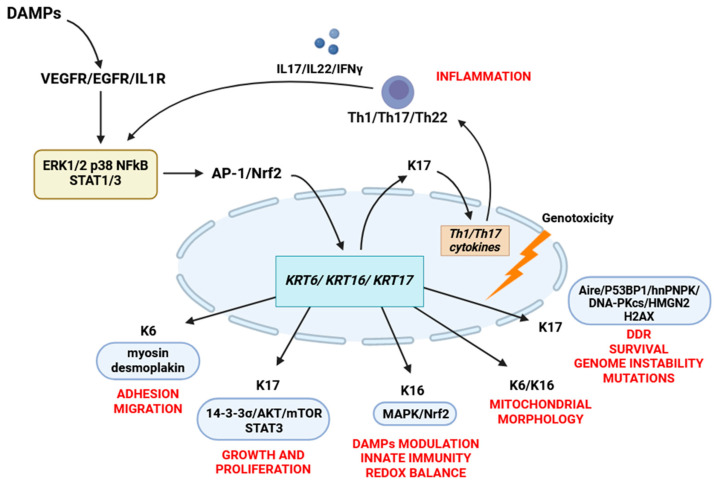
The cell functions of K6, K16, and K17. Damaged keratinocytes release various DAMPs, which induce the expression of K6/16/17 by activating the AP-1, NFkB, Nrf2, MAPK (ERK1/2 and p38) pathways. Altered expression of K17 promotes the secretion of Th1 cytokines (IFNγ, IL17A, and IL22) that maintain K6/16/17 expression through an autoimmune feedback loop. Keratin 17 regulates cell growth and proliferation by binding to the 14-3-3σ/AKT/mTOR pathway and STAT3 phosphorylation. When located in the nucleus, keratin 17 is involved in the DNA damage response by interacting with various DNA damage response proteins, including Aire, hnRNPK, H2AX, DNA-PKcs, 53BP1, and HMGN2. Keratin 16 modulates the secretion of DAMPs through the MAPK and EGFR signaling pathways and plays a role in maintaining the redox balance by interacting with the Nrf2 signaling pathway. Keratins K6 and K16 also play a role in regulating the morphology and functions of mitochondria. Keratin 6 is involved in regulating cell adhesion by directly interacting with myosin IIA and desmosomal proteins, which provide the mechanical properties necessary for wound healing.

The currently known functions of keratin 6 mainly involve regulating keratinocyte migration through enhancing cellular adhesion and attachment to the substrate. This is achieved through the interactions between keratin 6a/6b and myosin II and desmoplakin [37]. However, there is still potential for discovering new and unexpected roles for keratin 6. Studies have shown that the addition of recombinant keratin 6A to cell culture media can slow proliferation and stabilize stem cells while also inducing the expression of stem cell-related genes such as *CD133*, *OCT4*, *KLF4*, *SOX2*, *NANOG*, and *ANAX2* [124]. The cell functions of K6, K16, and K17 are illustrated in Figure 2.

## 8. Regulation of K6, K16, and K17

One of the common properties of all cytokeratins is the ability of their promoters to bind various transcription factors. Among these transcription factors, those involved in the regulation of KRT6/16/17 include proteins from the AP-1 and NFκB signaling pathways. Specifically, in cooperation with Sp1, AP-1 can activate the promoters of *KRT6* and *KRT16* [125,126]. Considering that the AP-1 and NFκB response elements can be physically separated, it is assumed that the interaction between these signaling pathways regulates the expression of epidermal genes. Activation of these factors leads to the differential expression of cytokeratins during epidermal differentiation, wound healing, and skin diseases.

Another transcription factor that regulates *KRT6*/*KRT16*/*KRT17* expression is Nrf2 [47,127]. Nrf2 regulates the expression of keratins 6, 16, and 17 by binding to specific regions of their promoters. These regions, known as ARE (antioxidant response element) domains, are located within the DNA sequences encoding these keratins [47]. Nrf2 is highly expressed in various types of skin cancer and other diseases. Its increased regulation can lead to the hyperproliferation of keratinocytes by inducing the expression of keratins 16 and 17 [47,127]. All three keratins are transcriptionally activated by the Hedgehog-regulated factors GLI1 and GLI2, suggesting a connection between these keratin proteins and the Hedgehog signaling pathway [128,129,130].

Some members of the p53 family of transcription factors are also involved in the regulation of K6/K16/K17. P53 is essential for the normal differentiation of epithelial cells [131]. The mechanisms by which p53 affects keratin are complex and involve both the activation and suppression of genes. p53 can indirectly suppress the expression of Krt14 in basal keratinocytes, contributing to their differentiation [132]. However, a recent study showed that removing p53 promotes differentiation by partially regulating the expression of *KRT6A* [133]. Therefore, the effect of p53 is complex and multifaceted. It directly binds to the promoter region of the *KRT17* gene and negatively regulates its expression. However, the induction of *KRT17* expression does not depend on the presence of p53 [134]. Additionally, the promoter sequence of *KRT17* contains two p53 response elements [134].

Other important factors involved in the regulation of K6/16/17 include cytokines and growth factors. Moreover, the initial activation of these proteins is carried out autonomously by keratinocytes. Damaged keratinocytes secrete a variety of DAMPs, such as dsRNA, DNA, high mobility group box 1 (HMGB1), uric acid, and heat shock proteins (HSPs) [135,136,137]. These molecules are recognized by pattern recognition receptors (PRRs) such as Toll-like receptors (TLRs). This leads to the activation of AP-1 and NFkB, which, in turn, activate *KRT6*/*16*/*17*. The expression of these keratins triggers the secretion of cytokines (IFN, IL17A, and IL22), which, in turn, support the expression of K6, K16, and K17 [2,37,43,128,134,138]. Epidermal growth factor (EGF) induces the expression of keratin on 6/16/17 through the NFκB and/or MAPK (ERK1/2) pathways [139,140,141]. TGF-β also activates the expression of keratin 17 in normal keratinocytes [17].

The expression of the K6, K16, and K17 genes can also be regulated in response to various external stimuli such as mechanical stimuli (stretching and scratches). These stimuli lead to the phosphorylation of the EGFR and ERK1/2 proteins, which, in turn, activate the expression of keratin 6 and repress the expression of K10 [142]. The expression of keratin 17 is induced by ionizing radiation (IR), and high levels of keratin 17 are characteristic of IR-induced dermatitis [134]. Keratin 17 is also induced by UV radiation from both UVA and UVB sources [143,144]. Our group reported that the expression of keratin 17 significantly increases in primary human keratinocytes exposed to cadmium in vitro [145]. Notably, a similar response was observed in HaCaT cells with knocked-out mutant p53, but not in wild-type HaCaT cells [146].

## Figures and Tables

**Table 1:** Expression of K6/16/17 in various types of cancer.

Cancer Type	Gene			References
	*KRT17*	*KRT16*	*KRT6*	
Oral squamous cell carcinoma (OSCC)	+	+	+	[13,87]
Lung adenocarcinoma (LUAD)	+	+	+	[15,80,84]
Invasive breast carcinoma (BRCA)	+/−		+	[88,89]
Ovarian cancer	+		+	[90,91]
Urothelial carcinoma (UCC)		+	+	[92]
Bladder cancer			+	[93]
Gastric cancer	+	+		[78,94]
Pancreatic ductal adenocarcinoma (PDAC)	+	+		[81,95,96]
Esophageal cancer (ESCC)	+	+		[97,98]
Tylosis with esophageal cancer (TOC)		+		[98]
Hypopharyngeal squamous carcinoma (HSCC)	−			[99]
Colorectal cancer (CRC)	+		+	[82,86]
Lung squamous cell carcinoma (LSCC)	+			[80]
Cervical cancer (CC)	+			[100]
Endometrial carcinoma	+			[74]
Chromophobe renal cell carcinoma (KICH)	+			[16]
Papillary renal cell carcinoma (KIRP)	n.d.			[16]
Pancreatic adenocarcinoma (PAAD)	n.d.			[16]
Thyroid cancer	+			[101]
Basal cell carcinoma (BCC)	+			[102]
Basaloid follicular hamartoma (BFH)	+			[102]
Head and neck squamous cell carcinoma (HNSCC)	+			[79,103]
Cutaneous squamous cell carcinoma (cSCC)	+	+	+	[104,105,106]

Symbol explanation: “+”—upregulated, “−”—downregulated, “n.d.”—no difference between normal and cancer tissue).

## Data Availability

No new data were created or analyzed in this study.
